# Incipient tuberculosis: a comprehensive overview

**DOI:** 10.1007/s15010-024-02239-4

**Published:** 2024-04-08

**Authors:** Salvatore Rotundo, Maria Teresa Tassone, Francesca Serapide, Alessandro Russo, Enrico Maria Trecarichi

**Affiliations:** 1 Department of Medical and Surgical Sciences, “Magna Graecia” University, Catanzaro, Italy; 2 Infectious and Tropical Disease Unit, “Renato Dulbecco” Teaching Hospital, Catanzaro, Italy

**Keywords:** Tuberculosis, Incipient tuberculosis, Tuberculosis infection

## Abstract

In the context of the evolving global health landscape shaped by the COVID-19 pandemic, tuberculosis (TB) is gaining renewed attention as a reemerging threat even in low-endemic countries. Immunological tests such as the tuberculin skin test (TST) and interferon-gamma release assay (IGRA) are pivotal in identifying tuberculosis infection (TBI). However, their inability to distinguish between past and ongoing infection poses a diagnostic challenge, possibly leading to the unnecessary treatment of a significant portion of the population with potential side effects. This review delves into the concept of incipient tuberculosis (ITB), a dynamic, presymptomatic stage characterized by heightened *Mycobacterium tuberculosis complex* (MTC) metabolic activity and replication that result in minimal radiological changes, signifying a transitional state between TBI and TB. Key focus areas include epidemiological factors, underlying pathogenesis, imaging findings, and the ongoing challenges in the identification of individuals with ITB through the development of new biomarkers and the use of whole-genome sequencing-based analyses to implement early treatment strategies.

## Introduction

The *Mycobacterium tuberculosis complex* (MTC) is still the leading cause of death for single infectious agent after SARS-CoV-2 and following geopolitical changes due to the coronavirus disease 2019 (COVID-19) pandemic and war in high-endemic territories (*e.g.*, Eastern Europe, sub-Saharan Africa). For these reasons, tuberculosis (TB) could be configured as a reemerging disease even in low-endemic countries in the next future [1]. In this scenario, the understanding of the natural history of MTC infection to diseases is improving, and a state of early and not contagious disease has recently been considered [2–4].

Detection of immunoreactivity against MTC has been made possible through tests such as the tuberculin skin test (TST) or interferon-gamma release assay (IGRA), revealing a staggering two billion individuals worldwide—equivalent to a quarter of the global population—supposedly infected with MTC [5]. Despite this prevalence, the majority of individuals exhibiting immunoreactivity successfully clear the infection without progressing to active TB. However, neither TST nor IGRA prove specific enough to differentiate between past (*i.e.*, cleared or treated TB), latent or ongoing infection [6]. In this context, the conventional term “latent tuberculosis infection (LTBI)” is being substituted with “tuberculosis infection (TBI),” according to the recent definition by the World Health Organization (WHO) [7], to better reflect the dynamic nature of this condition. Indeed, TBI describes a state characterized by persistent immune response to MTC antigens without clinically manifesting TB disease. This updated definition highlights the evolving understanding of TBI and the omission of “latent” acknowledges that tuberculosis infection is not always dormant, aligning with current insights into its complex pathogenesis [7]. However, the definition provided by the WHO of TBI encompasses a spectrum of states, without clearly delineating the distinctions between TBI and incipient tuberculosis (ITB). Indeed, TBI is an ambiguous concept as it encompasses a spectrum of different stages that may or may not progress to active TB. In contrast, ITB denotes a condition in which people infected with MTC are likely to evolve to TB without therapeutical intervention, even though both TBI and ITB remain clinically silent and undetectable through traditional microbiological techniques [3]. This comprehensive framework recognizes the complexity inherent in TBI dynamics, incorporating both the latent and incipient stages, and underscores the need for tailored approaches in diagnosis and management.

At this stage, even the most sensitive microbiological tests are not able to detect traces of the MTC in any biological sample, though the assumption remains that the MTC may be isolated through culturing lung biopsies due to the viability of bacilli [6, 8]. From a pathophysiological perspective, TBI represents a dynamic equilibrium between the MTC and the host immune system. Indeed, it is not expected to evolve into TB unless significant immunocompromising conditions occur, as the host immune system effectively controls the replication of MTC, preventing the infection from spreading [3, 9, 10].

TBI remains a post hoc diagnosis, encompassing individuals who, despite persistent immunoreactivity against MTC, do not progress to TB. Clinically, TBI is often diagnosed through the exclusion of TB [6, 9, 11]. Importantly, individuals with TBI cannot transmit the infection to others unless they progress to TB. Notably, only a minority of people with immunoreactivity against MTC experience progression to TB during their lifetime, accounting 5–10% of untreated cases overall [3, 9, 12]. Therefore, a substantial proportion of these people may receive unnecessary treatment, burdened by potential significant side effects [13, 14].

## Proposed definitions

Some individuals infected with the MTC fail to restrain its replication, progressing to TB without exhibiting any overt signs or symptoms. This condition, known as incipient tuberculosis (ITB), was proposed by Achkar JM et al. [2] to define the combination of radiological findings affecting the upper pulmonary lobes exceeding 2 cm^2^ in size in apparently immunocompetent individuals with a history of prior TB exposure. However, this definition may be inaccurate since a fraction of these cases could be categorized as affected by subclinical TB since viable MTC could be detectable through real-time polymerase chain reaction (RT-PCR) in highly sensitive samples such as bronchoalveolar fluid (BALF) [15]. Moreover, the specificity of upper pulmonary lobe lesions in detecting ITB can be insufficient, particularly in settings where immunoreactivity against MTC is prevalent. Importantly, both chest X-ray (CXR) and computed tomography (CT) lack the necessary sensitivity to predict the progression to TB in individuals with TBI [16].

Drain PK et al. grappled with the nuanced definitions of ITB across various authors [3]. Their article aimed not only to reconcile these differences but also to delve deeper into the early stages and progression of infection, shedding light on the subtleties and complexities of this crucial aspect of TB pathology. Indeed, in a more detailed way, ITB may be defined as a transitional, presymptomatic clinical condition characterized by the presence of viable bacilli that evade control by the host immune system that typically progresses to TB without early therapeutic interventions although, at this stage, transmission to other people is improbable [3]. In such cases, immunoreactivity against MTC can usually be demonstrated through TST or IGRA [6] and, despite evidence for TB is not demonstrable with routine tools for screening (*i.e.*, CXR and sputum microbiology) [17], MTC hematogenous seeding can be evidenced through recently developed high-throughput tests [4, 18, 19]. These are nucleic acid-based biomarkers that include specific DNA [4, 18, 19] or RNA [20] sequences associated with the presence of the MTC. RT-PCR, sequencing, and bacteriophage-guided nucleic acid amplification techniques are proposed strategies employed to detect and amplify these putative genetic markers.

Despite TB being a continuum of infection that can remain latent within the host for several years [21], recent pathophysiological developments have sparked endeavors to comprehend specific clinical conditions (Table 1) and comprehending ITB may present a chance both for the development of novel diagnostic strategies and early treatment of the individual, optimizing resources, mitigating unnecessary treatments, and contributing to the overall improvement from a public health perspective by reducing the risk of MTC transmission.

**Table 1:** The pathological spectrum of latent tuberculosis infection

	Clinical findings	Imaging findings	Laboratory findings
Cleared tuberculosis infection^*^	None	None	None
Tuberculosis infection		Pauci-nodular lesions [22] or scars/infiltrates usually involving upper pulmonary lobes [18]	MTC might be demonstrated by culturing infected tissue [8]
Incipient tuberculosis		Pauci-nodular lesions or scars/infiltrates usually involving upper pulmonary lobes [22] with increased ΔSUVmax [23]	MTC might be demonstrated by culturing infected tissue [8] Blood DNA signatures [4, 18, 19] could be detected on blood samples
Subclinical tuberculosis		Parenchymal opacities, cavitation, lymphadenopathy, and/or pleural effusion	MTC can be demonstrated with culture, PCR, or microscopy on respiratory samples
Tuberculosis	Prolonged cough, chest pain, weakness, fatigue, weight loss, fever, night sweats	Parenchymal opacities, cavitation, lymphadenopathy, and/or pleural effusion	MTC can be demonstrated with culture, PCR, or microscopy on respiratory samples

In such cases, *M. tuberculosis complex* (MTC) is usually demonstrable via tuberculin skin test (TST) or interferon-gamma releasing assay release assay (IGRA)

*PCR* polymerase chain reaction, *PET* positron emission tomography, *ΔSUVmax* delta maximum standardized uptake value

^*^It refers to MTC infection cleared by the immune system of the host without any therapeutic intervention

## Epidemiology and pathogenesis

The epidemiology of ITB is dynamic and influenced by various factors, including public health interventions (*i.e.*, targeted testing, contact tracing, treatment of individuals with TBI at higher risk of progression to TB) and social determinants including poverty, malnutrition, and lack of access to health-care system [12]. Indeed, the real prevalence of ITB is unknown and difficult to estimate for several reasons. First, there is no consensus on ITB definition [3]. Second, ITB is a subclinical condition which is not detectable considering symptoms and radiological or microbiological findings. Third, there are no validated diagnostic tests for ITB. Fourth, diagnosis of ITB in some immunocompromising patients—particularly those with T cell impairment—could be tricky since TST and IGRA are more likely to test negative [6].

However, it was estimated that, among people who are infected with MTC, 56 million could progress to TB [13]. Interestingly, Dye et al. [24], who utilized mathematical modeling techniques to derive perspectives for TB elimination. estimated that if 8% of people with TBI could be identified and treated, there would be a 14-fold reduction in TB incidence without any additional intervention. This further underscores the potential effectiveness of targeted TBI treatment as a critical strategy for reducing the global burden of TB and achieving ambitious public health goals.

Comprehending the pathogenesis of ITB is crucial for developing strategies to prevent the progression to TB. However, this understanding necessitates delving into intricate and not fully elucidated interactions between the MTC and the host immune system. However, investigating the molecular and immunological aspects of ITB, exploring strain-specific interactions, and understanding the impact of aging and comorbidities on TBI progression could pave the way for innovative strategies for prevention, diagnosis, and early treatment of TB.

In the early phase of the primary TBI, bacilli are phagocyted by host immune cells that reach the lung tissue from bloodstream to clear infection. However, these cells become infected and fail to kill bacteria since the MTC can replicate inside phagocytes [5]. Indeed, bacteria quickly reprogram their metabolic activities, switching to anaerobic respiratory pathways into intracellular environment [25]. Moreover, modern MTC phylogenetic lineages replicate faster and elicit a weaker and delayed inflammatory response than ancient and intermediate lineages. The term “modern” is collectively used to describe those strains that undergone genetic diversification more recently compared to the “ancient” and the “intermediate” lineages. Modern MTC phylogenetic lineages arose from a shared ancestor after the loss of the MTB-specific deletion 1 region (TbD1), which encodes MmpS6/MmpL6 [3, 26]. Bottai et al. [26] demonstrated that these more recent strains show a decreased susceptivity to oxidative burst (*i.e.*, the rapid release of reactive oxygen species from phagocytes). This diminished susceptibility may play a pivotal role during infection in the intracellular environment [26]. Mycobacteria that are released after phagocytic cell killing recruit other phagocytic cells, promoting further bacterial replications [5]. Moreover, MTC inhibits neutrophils’ apoptosis. Importantly, because of these delay in adaptative immunity development, it becomes measurable approximately 6 weeks after infection using tests like TST or IGRA [6].

Mycobacteria are phagocyted by dendritic cells and transported from lungs to lymphoid tissues where they stimulate adaptative immunity [5]. In particular, T cell immunity assumes a central role in countering MTC, as highlighted by various studies [5, 27], and optimal Th1 and Th17 immune responses are identified as critical for sustaining long-term control of TBI, emphasizing the importance of a balanced Th1/Th2/Th17 response to mitigate tissue pathology [28]. Notably, the Th1 response emerges as particularly advantageous in the fight against MTC, suggesting a path for further research into strategies that enhance these immune responses. Indeed, contrary to a prominent antibody-mediated response to MTC which is known to be associated with progression of TB [27], MTC-specific T cells align with the arrest of bacterial proliferation, generating TNFα and interferon-gamma (IFNγ) to increase macrophage activity [28]. However, despite effective in controlling the infection, these mechanisms exhibit limitations in definitively clearing MTC [5]. Therefore, on one hand, the immune cells attempt to isolate and control the infection by forming granuloma, playing a complex role in defending the host against TBI, but, on the other hand, they can also contribute to tissue damage and pathology [28]. With regard to these aspects, PLWH seems to be at higher risk of TB progression since the early stage of HIV infection (*i.e.*, before a measurable reduction of CD4 + T cells) because of both qualitative and quantitative deficit of T cells [5, 25]. In such cases, the classical granulomatous inflammation appears to be significantly different with higher burden of bacilli and lower reactive features [2]. The heightened susceptibility of PLWH to TB progression and the altered granulomatous inflammation in these individuals underscore the need for exploring the intricate crosstalk between HIV and MTC. Investigating how HIV-induced T cell deficits contribute to the altered immune response in ITB and identifying potential targets for restoring immune functionality could be a promising avenue although the primary measure to prevent the reactivation of TB in these patients remains antiretroviral treatment.

In patients who reactivate TBI, mycobacteria increase their metabolic activity [3]. The ambiguity surrounding whether the heightened metabolism results from an escalation in bacterial virulence or a compromised ability of the host to control the infection presents a complex and intriguing aspect of the pathophysiology. One plausible path is the notion that the bacteria might undergo adaptive changes leading to an increase in virulence. This could involve genetic mutations or alterations in bacterial physiology, enabling them to evade host defenses more effectively or manipulate the host environment to their advantage. The evolution of more virulent strains could potentially contribute to the exacerbation of the infection, posing challenges for the host’s immune system [3]. On the contrary, an alternative speculation revolves around the host’s immune response and its potential decline in effectiveness over time. Factors such as immune exhaustion [5], immunosenescence (*i.e.*, age-related weakening of the immune system), or the influence of comorbidities and immunosuppressive treatments might compromise the ability of the host to mount an efficient defense against the progressing infection [12]. For instance, chronic inflammation may disrupt the granulomas formed during the initial infection, providing MTC an opportunity to escape and replicate. Moreover, reactivation of TBI is promoted by the therapeutic blocking of TNFα [5, 28]. However, ITB could affect any person infected with MTC since all the risk factors listed above including both regarding host (*e.g.*, HIV infection, immunosenescence, therapeutic blocking of TNFα) and MTC (*e.g.*, phylogenic lineage) explain only a fraction of cases with TB progression or reactivation [5]. This scenario raises questions about the resilience and adaptability of the immune mechanisms in the face of a persistent and evolving threat. Unraveling whether the observed escalation in infection severity is primarily driven by bacterial factors, host-related factors, or a synergistic combination of both will be crucial for informing targeted interventions and therapeutic strategies.

## Imaging findings

The radiological features of TB typically manifest as parenchymal opacities, cavitation, lymphadenopathy, and/or pleural effusion [16]. Notably, in the early stages of TBI, chest X-rays (CXR) yield negative results in 25–40% of cases [16].

Interestingly, a systematic review and meta-analysis conducted by Sossen et al. [10], who aimed to quantify the rates of progression and regression across the TB disease spectrum, found an annualized progression rate of approximately 10% in individuals displaying active radiographic TB changes. Conversely, patients with either normal baseline CXR or those displaying inactive or fibrotic changes demonstrated markedly lower progression rates, ranging from 0–1%. Moreover, CXR tends to be consistently negative in individuals with ITB [22]. Therefore, despite CXR remaining a cornerstone in diagnosing pulmonary TB [16], its efficacy diminishes in detecting the subtle changes associated with ITB [22].

Computed tomography (CT) stands out as a more sensitive imaging modality compared to CXR when it comes to identifying parenchymal lesions associated with TBI and detecting lymphadenopathies. This heightened sensitivity is particularly beneficial in cases of ITB since minimal morphological changes may not be easily discernible on conventional CXR. Indeed, the CT images could reveal early pauci-nodular lesions or scars/infiltrates usually involving upper pulmonary lobes even in patients who test negative on molecular test on BALF [22].

It is crucial to note that while CT is apt at revealing morphological alterations, interpreting these changes in terms of MTC activity poses a substantial challenge. The presence of granulomas, a common finding in CT scans, is not necessarily indicative of active TB. In many instances, granulomas signify a resolved MTC infection or a state of TBI rather than an ongoing active disease [16].

Therefore, despite its ability to detect morphological changes, distinguishing between active and inactive lesions on CT scans remains intricate. The challenge lies in accurately assessing the dynamic status of these lesions, whether they represent ongoing TB activity or residual changes from prior infections. This nuanced aspect emphasizes the need for complementary diagnostic tools and a comprehensive clinical evaluation to determine the true nature of the observed morphological alterations on CT scans.

In such scenarios, positron emission tomography (PET) becomes a valuable tool for assessing metabolic activity, revealing functional changes attributed to heightened metabolism in pulmonary lesions [16, 22, 23, 29]. This increased metabolic activity [30] serves as an indicator of ongoing or impending progression to TB [3]. Kim et al. [23] have proposed the delta maximum standardized uptake value (ΔSUVmax) as a potential predictor for the activity of pulmonary tuberculoma. This metric demonstrates a high rate of both sensitivity and specificity, effectively distinguishing between active and inactive pulmonary lesions. By contrast, a recent prospective study by Kim et al. [17] including healthy, immunocompetent individuals who had household contact with pulmonary TB patients downplayed the role of PET scan in the diagnosis of ITB. Indeed, 2/10 (20%) individuals identified as baseline PET positive contacts exhibited progressive features upon their subsequent scan performed 3 months later. This finding suggests that the sole reliance on a single PET scan may prove inadequate in terms of specificity for detecting ITB [17].

Therefore, the interpretation of PET findings is challenging. Furthermore, the fluorodeoxyglucose used in PET scans is captured not only by MTC undergoing active replication but also by immune system cells attempting to contain the bacilli. Given the fluctuating metabolic activity of MTC during infection, which alternates between quiescence and heightened activity, the predictive value of PET in detecting patients with ITB remains unclear. In addition, the considerable cost associated with PET limits its widespread use, particularly in low-income countries [29]. Furthermore, especially in high-endemic countries, PET may be unable to distinguish a solitary malignant nodule from a tubercular one [16].

In the context of ITB, PET holds potential in assessing the response to antibiotic treatment [16], although its definitive role in this regard is yet to be established. Continued research is needed to validate the utility of PET in monitoring clinical evolution and treatment responses and its feasibility in diverse health-care settings, taking into account both clinical efficacy and economic considerations.

## Advances in molecular testing

The WHO and the Foundation for Innovative New Diagnostics have established criteria for new tests designed for TBI, stipulating that they should demonstrate both sensitivity and specificity ranging from 75 to 90% [31]. Currently available clinical tests meet these criteria, but they are not without drawbacks, often yielding a significant number of false-positive and false-negative results that impact their ability to accurately predict a prior TBI [6].

For instance, the TST can be influenced by factors such as prior Bacillus Calmette–Guérin vaccination and exposure to nontuberculous mycobacteria, affecting its specificity [11]. IGRA and TST may test negative patients with disseminated TB [6]. Both TST and IGRA may also produce false-negative results in recent contacts of patients with pulmonary TB and in immunocompromised patients, whereas false-positive results are expected in individuals who have successfully cleared MTC. Importantly, achieving a balance between accurately detecting cases and minimizing false positives or negatives remains an unmet need in ITB testing. Indeed, although several studies have provided evidence of a correlation between the magnitude of both TST [32] and IGRA [33] and the risk of progression to TB, one of the foremost challenges in clinical practice remains to accurately identifying individuals with TBI infection who are most likely to progress to active TB disease. Several studies have demonstrated that a meticulous assessment of risk factors can substantially reduce the number needed to treat (NNT) to remarkably low levels [29]. Through ongoing research and refinement of risk assessment methods, health-care providers can continue to improve TB prevention efforts and mitigate the burden of this infectious disease.

Certain molecular tests have shown promise in identifying patients infected with ITB even in people without major immunocompromising conditions. For instance, a blood RT-PCR test that detects MTC DNA after bacteriophage-mediated lysis of MTC cells exhibited very high specificity in predicting ITB, especially among patients with immunoreactivity against MTC who reported recent contact with individuals affected by pulmonary TB [4]. As for this matter, a positive correlation between a baseline test yielding positive results and the subsequent receipt of TB treatment was observed during the prospective follow-up [17]. Another transcriptomic signature identified through RNA sequencing, characterized by an overabundance of interferon-inducible genes and a scarcity of genes of both B and T cells, demonstrated robustness in distinguishing TB patients from individuals with TBI and healthy subjects [20]. However, gene signatures, particularly those dominated by IFN-inducible genes, may lead to potential false positives in the presence of viral infections [20].

The exploration of molecular tests for ITB diagnosis, particularly in immunosuppressed patients who may test negative in traditional immunological tests, has garnered interest. Regarding this topic, a 16-gene blood transcriptional signature demonstrated a sensitivity of 66.1% and a specificity of 80.6% in predicting TB occurrence in a cohort of PLWH [18].

**Fig. 1:**
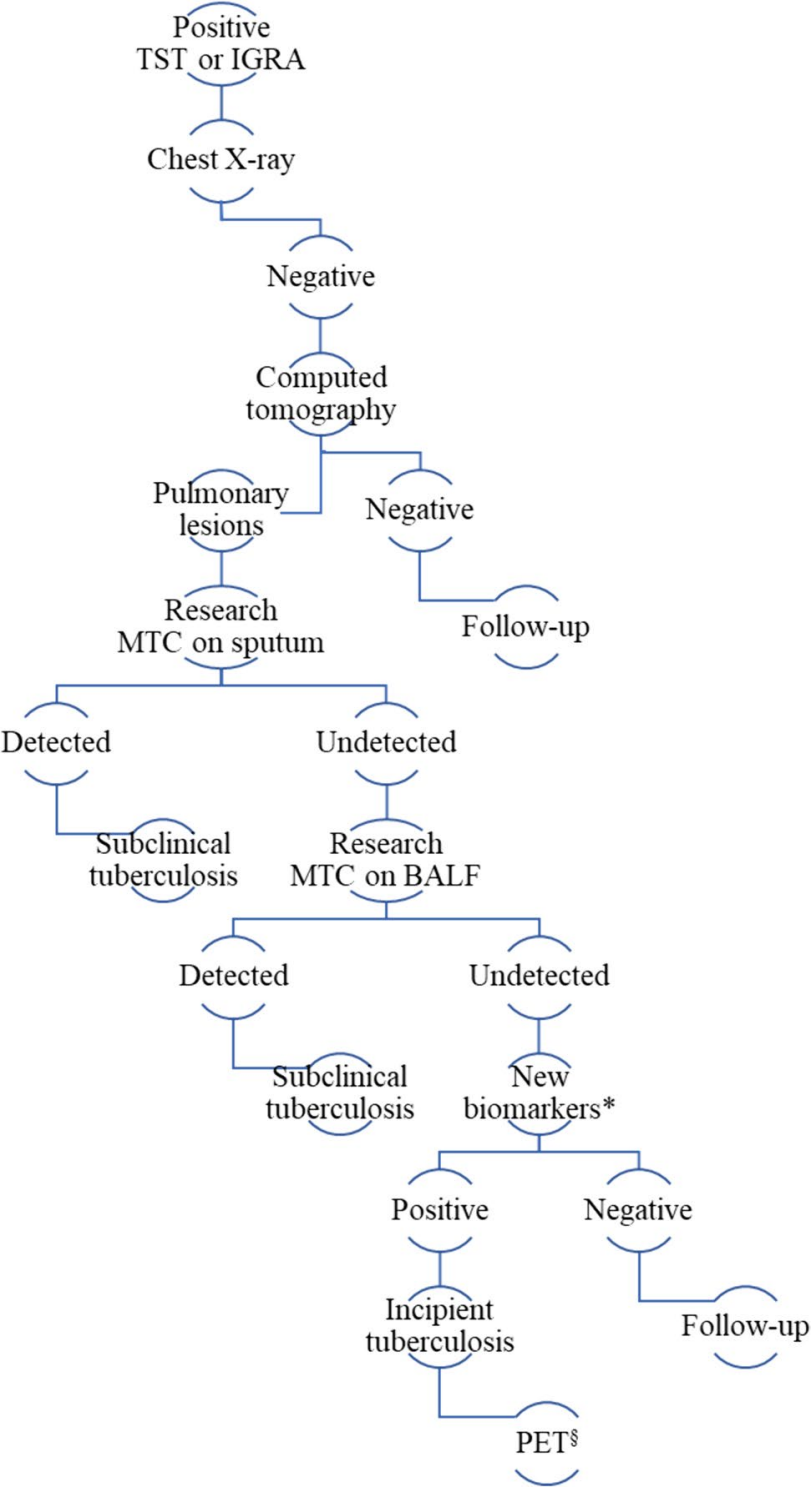
Proposed algorithm in asymptomatic patients with immunoreactivity against *M. tuberculosis complex* (MTC) demonstrated trough tuberculin skin test (TST) or interferon-gamma release assay (IGRA). *BALF* bronchoalveolar lavage fluid, *PET* positron emission tomography. *Traces of the MTC genome detectable by high-throughput techniques on blood samples, details in the text. ^§^Consider PET for monitoring response to treatment in patients with incipient tuberculosis (ITB), details in the text.

Since these candidate tests demonstrated high specificity but low sensitivity, a serial testing strategy, such as testing every semester for the first 2 years from the detection of immunoreactivity against MTC, may be required. However, the high cost and limited availability of these molecular tests could pose challenges for implementing this strategy as screening at a population level in the next future [34]. Conversely, serial blood testing for these candidate tests within the first 2 years from selected patients with features suggesting ITB might be more feasible (Fig. 1). This approach could be particularly relevant for recent or household contacts, migrants from high-endemic countries, and individuals with radiological findings suggestive of lesions due to TBI despite negative microbiological evidence [34].

Interestingly, although the widespread use of signatures as a screening test for ITB is limited, several molecular tests show adequate sensitivity in the 3 months preceding TB diagnosis and is still unclear whether their application in selected groups of patients at high risk of progression could prove valuable for early diagnosis and shorter treatment periods [34].

## Treatment

Patients identified with ITB could benefit from early intervention to prevent the progression to TB [2]. In addition, the lower bacterial burden associated with early-stage infections may render them more amenable to easier treatments compared to those used for TB [3]. However, the absence of a clearly defined diagnostic framework has hindered the assessment of targeted treatment regimens for this distinct subgroup.

Engaging in speculative reasoning, conventional regimens commonly employed for TBI could potentially extend their efficacy to ITB cases, assuming a relatively modest disease burden [3]. For instance, isoniazid and rifapentine administered once a week for a duration of 3 months, daily rifampin for a period of 4 months, the combination of daily isoniazid and rifampin spanning 3 to 4 months, and a regimen involving daily isoniazid over a course lasting 6 to 9 months that are recommended for TBI treatment in high-risk patients [13] might be effective in ITB. It is noteworthy, however, that a preferred approach involving a 3-month course of once-weekly, high-dose isoniazid and rifapentine, recommended for the treatment of TBI [9], demonstrated ineffectiveness in reducing progression to TB over a 15-month period in HIV-negative patients diagnosed with ITB through a blood transcriptomic molecular signature [31]. Importantly, this test had been previously validated solely for diagnostic purposes in PLWH.

Furthermore, it is essential to take into account the potential for drug resistance in this empirical approach. Recognizing the variability in individual responses to treatment and the evolving landscape of the MTC, considering the specter of drug resistance becomes a pivotal aspect of any comprehensive strategy for managing ITB. In this regard, a significant drawback of this preliminary methodology lies in the potential unavailability of an antibiogram, particularly given the inherent challenges in isolating MTC through culture methods in the case of ITB, unless a biopsy is conducted. However, an intriguing alternative arises as MTC DNA can be successfully extracted from the blood of individuals afflicted with ITB [18, 19]. Of note, the employment of whole-genome sequencing-based analyses for assessing MTC resistance has demonstrated interesting results. Indeed, these analyses have exhibited a remarkable level of agreement with traditional drug susceptibility testing for the MTC, particularly in the identification of drug resistance against key antituberculosis medications such as rifampin, isoniazid, pyrazinamide, and quinolones [35]. This convergence in outcomes underscores the reliability and validity of whole-genome sequencing as a diagnostic tool, providing a feasible substitute when the conventional antibiogram might be inaccessible due to the challenges in culturing the MTC from ITB patients, thus contributing to a more comprehensive and effective strategy for choosing the most suitable treatment for ITB.

This underscores the complexity of tailoring treatment strategies for different patient populations and the need for further research to refine and expand our understanding of the optimal therapeutic approaches for individuals with ITB.

## Conclusion

ITB emerges as a discrete and transitional clinical condition characterized by an elevation in MTC metabolic activity, attributed to an inadequate immune system control. The precise prevalence of ITB remains elusive, and while several factors are linked to TB progression, they account for only a minority of cases. Consequently, ITB could potentially affect any individual infected with MTC, necessitating further investigations to identify those most at risk.

The pathogenesis of ITB involves intricate and largely unknown interactions between the host system and bacteria, with T cells potentially playing a pivotal role. Unlike typical TB presentations, CXR is typically negative in most patients with ITB, and CT reveals minimal changes. On the contrary, PET proves highly sensitive in detecting metabolic changes associated with ITB. However, the limitations of low specificity and cost impede their widespread use, particularly in low-income countries.

Currently, no single test has attained sufficient accuracy for diagnosing ITB. Consequently, until the predictive capacity of molecular tests is established, a comprehensive assessment of ITB should rely on a combination of clinical, laboratory, and imaging findings. This multifaceted approach ensures a more holistic understanding of ITB and aids in devising effective strategies for its diagnosis and management.
